# Comment on “Rationale and design of a pilot randomized control trial of permissive hypotension after cardiac surgery: The PHACS trial”

**DOI:** 10.1016/j.ahjo.2026.100948

**Published:** 2026-10-08

**Authors:** T. Kalpana, C. Silpa, Manjusha Manukonda, A. Ponmani

**Affiliations:** a College of Nursing, Sri Padmavati Mahila Visvavidyalayam Women's University, Tirupati, India; b Department of Community Health Nursing, Apollo College of Nursing, Chittoor, India; c West Godavari District, Andhra Pradesh; d Vikram College of Nursing, Madurai, 625020, India

Dear Editor,

Miranda et al. [1], Describe the PHACS pilot trial, which randomizes patients needing vasopressors after surgery to either a mean arterial pressure (MAP) target of 60 to 65 mmHg or to usual care. The question is important. The pragmatic design makes sense. Still a few parts of the protocol might affect how we interpret the results.

The statistical plan does not seem to match the sample size. The null hypothesis states that the between-group difference in patient-averaged MAP is than 5 mmHg, tested with a one-sided alpha of 0.025. In this case, 5 mmHg is the margin that must be exceeded to reject the null hypothesis [2]. A trial powered at 80% to detect a difference of exactly 5 mmHg would barely have power to reject it. Also no standard deviation for MAP is given. That means readers can't check how the authors got to 40 patients per arm.

The primary outcome and time window require definition. Randomization requires at least 1 h of vasopressor therapy, but the analytic window starts when vasopressors are initiated [3]. Which the authors say “corresponds to randomization.” The window ended after 24 h without vasopressor administration. Outcomes were measured during vasopressor therapy. If the intervention shortens the duration of vasopressor therapy, as the study expects, each group will have observation times. In addition, the Methods mention using readings even though every patient on vasopressors is supposed to have an arterial catheter.

Achieving separation between groups may be more difficult than expected. Hypotension for randomization is defined as MAP < 65 mmHg, requiring vasopressors to maintain MAP above 65 mmHg. This suggests that 65 mmHg is already the standard of care. This is also the upper limit of the permissive range. Therefore, a 5 mmHg difference depends on clinicians in the control group aiming above 65 mmHg, which may not happen.

The plan for the randomization events was unclear. Patients started the assigned strategy only if they met the eligibility criteria. Also exit criteria. Similar to rising lactate levels, re-exploration, support, and cardioversion. This was only applied to the permissive arms. When patients exited, they returned to care. The protocol does not specify whether the analysis will be conducted by intention to treat or per protocol [4]. It also does not explain how exclusions after randomization will be handled.

Some other points require clarification. The trial is labeled, but the strengths section states that it is blinded to the patient. Logistic regression was proposed for binary outcomes to adjust for confounding. Randomization should already handle this. With 80 patients, there will not be many events. Safety oversight is handled by a committee of the principal investigators, not an independent board. This seems risky because the trial lowers blood pressure after bypass. Lastly, for a feasibility study, no progression criteria are given. Such criteria include the recruitment rate, proportion of consented patients who got randomized, and adherence thresholds.

Enrollment is now complete. Reporting the definitions and planned analysis population along with the results would help readers decide if the separation achieved and the safety record support moving to a larger multicenter trial.

## CRediT authorship contribution statement

**T. Kalpana:** Resources. **C. Silpa:** Conceptualization. **Manjusha Manukonda:** Project administration. **A. Ponmani:** Supervision.

## Informed consent

Not applicable.

## Ethical approval

Not applicable.

## Declaration of Generative AI and AI-assisted technologies in the writing process

Generative AI tools were used only for language refinement and formatting assistance. All scientific interpretation, critical appraisal, and manuscript preparation were performed independently by the authors.

## Funding

This research did not receive any specific grant from funding agencies in the public, commercial, or not-for-profit sectors.

## Declaration of competing interest

The authors declare that they have no known competing financial interests or personal relationships that could have appeared to influence the work reported in this paper.

## Data Availability

No new data were generated or analyzed in this study.

## References

[1] Miranda D.W., Makarem A., Liquori C. (2026). Rationale and design of a pilot randomized control trial of permissive hypotension after cardiac surgery: the PHACS trial. Am. Heart J. Plus.

[2] Ongaro A., Migliorati S., Ascari R., Ripamonti E. (2024). Testing practical relevance of treatment effects. Stat. Pap..

[3] Panwar R., Gibberd A., Oldmeadow C., Tiruvoipati R., Aneman A., Kansal A. (2025). REACT SHOCK trial protocol and analysis plan—a multicenter randomised controlled trial comparing individualised blood pressure target versus standard blood pressure target among critically ill patients with shock. Trials.

[4] Xie D., McKechnie T., Staibano P. (2025).

